# Mastery and self-esteem mediate the association between visual acuity and mental health: a population-based longitudinal cohort study

**DOI:** 10.1186/s12888-020-02853-0

**Published:** 2020-09-24

**Authors:** I. M. Maaswinkel, H. P. A. van der Aa, G. H. M. B. van Rens, A. T. F. Beekman, J. W. R. Twisk, R. M. A. van Nispen

**Affiliations:** 1 Amsterdam UMC, Vrije Universiteit Amsterdam, Ophthalmology, Amsterdam Public Health Research Institute, Amsterdam, The Netherlands; 2 Elkerliek Hospital, Ophthalmology, Helmond, The Netherlands; 3 Amsterdam UMC, Vrije Universiteit Amsterdam, Psychiatry, Amsterdam Public Health Research Institute, Amsterdam, The Netherlands; 4 GGZ inGeest Specialized Mental Health Care, Research and Innovation, Amsterdam, The Netherlands; 5 Amsterdam UMC, Vrije Universiteit Amsterdam, Epidemiology and Biostatistics, Amsterdam Public Health Research Institute, Amsterdam, The Netherlands

**Keywords:** Vision loss, Visual acuity, Depression, Anxiety, Mastery, Self-esteem, Longitudinal cohort

## Abstract

**Background:**

With deteriorating eyesight, people often become dependent on others for many aspects of their daily lives. As a result, they feel less ‘in control’ and experience lower self-esteem. Lower sense of mastery and self-esteem are known to predict depression, but their roles in people with visual impairment have only marginally been investigated. Therefore, this study aimed to determine the influence of mastery and self-esteem on the relationship between visual acuity and mental health.

**Methods:**

A longitudinal cohort study was performed using data from the Longitudinal Aging Study Amsterdam (LASA), collected between 2001 and 2012. A community-based population of 2599 older adults were included, who were randomly selected from population registers. Outcomes of interest were the Pearlin Mastery Scale, Rosenberg Self-Esteem Scale, Center for Epidemiologic Studies – Depression scale and the Hospital Anxiety Depression Scale – Anxiety subscale. Linear mixed models were used to establish the association between visual acuity and mental health over time.

**Results:**

Mean age was 72 years, 56% was female and 1.2% qualified as having low vision. Visual impairment was associated with a lower sense of mastery (β = − 0.477, *p* < 0.001), lower self-esteem (β = − 0.166, *p* = 0.008) and more depression (β = 0.235, p < 0.001). No significant association between visual acuity and anxiety was found. The relationship between visual acuity and depression was mediated by self-esteem (25%) and sense of mastery (79%).

**Conclusions:**

Vision loss was associated with depression. This association was mediated by self-esteem and sense of mastery. This provides us with new possibilities to identify, support and treat those at risk for developing depression by aiming to increase their self-esteem and sense of mastery.

## Background

Globally, about 285 to 440 million people suffer from visual impairment, mostly due to uncorrected refractive errors and cataract [1, 2]. Increasing life expectancy rates in high-income countries [3] are expected to dramatically raise the prevalence of visual impairment and subsequent need for eye-care services and associated health care costs for years to come [4]. Global health is also greatly impacted by mental health issues [5], especially in older adults [6] and in those with visual impairment [7–11]. The prevalence of depression in older adults with visual impairment is estimated to approximate a staggering 30%, compared to roughly 11% in control groups [12, 13]. And with an estimated prevalence of 15%, anxiety symptoms are twice as common in older adults with visual impairment than those without visual impairment [14].

Different mechanisms have been proposed to explain the association between visual impairment and mental health [15]. The severity of vision loss and coherent loss of functional capacity partly explain the association between visual impairment and mental health [16–20]. Social factors, i.e. supporting network size and social support, also seem to have an influence [11, 21]. However, many studies indicate that intrapersonal factors play a significant role in the psychological outcomes of vision loss [19, 22]. For instance, greater acceptance of vision loss seems to be an important predictor of improved mental health [19, 23–26]. In addition, problem solving skills [27], control strategies [28], (mal) adaptive coping strategies [22, 24], and a person’s perceived self-efficacy to use coping strategies [29] seem to largely influence mental health in people with vision impairment.

Two important intrapersonal factors, however, have not or only marginally been considered in previous studies. The first is mastery, which has been defined as the extent to which someone feels in control over his/her life and environment [30]. Several studies have shown positive associations of mastery with adaptation to stressful life situations and increased physical and mental health [31, 32]. As our society relies heavily on visual functioning, those with visual disabilities often experience a loss of control and dependency on others [33–35]. This experience of an ‘external locus of control’ [36] may be a major factor in the development of depressive symptoms in this population. The second factor is self-esteem, which has been defined as the way someone evaluates or appraises their own self-worth, and which is influenced by interactions with (significant) others [37]. Limitations in activities of daily living due to visual impairment, having to rely on others and facing negative attitudes towards visual disability, may largely influence a person’s self-esteem [35], which may increase their risk of mental health problems [38]. In a cross-sectional study, Kurtović et al. found that self-esteem was associated with depression in adults with visual impairment [21]. Besides optimism and social support, they emphasize the importance of focusing on self-esteem in rehabilitation practice to increase mental health in people with visual disability.

Because problems with mastery and self-esteem seem to increase after (completely or partially) losing vision [35], we propose that the relationship between vision loss and mental health is mediated by these intrapersonal factors. We hypothesise that visual impairment has a negative impact on mental health through a reduced sense of mastery and self-esteem. We aim to investigate this hypothesis in a large sample of older adults who were followed over time, which could provide us with greater insight into mental health in people with visual impairment, and help us determine new and better ways to address this problem.

## Methods

### Study design

A longitudinal cohort study was performed, using data from four time points from the Longitudinal Aging Study Amsterdam (LASA) [39], collected from 2001 through 2012. In this period, participants were measured four times; in 2001–2002 (cycle E), in 2005–2006 (cycle F), in 2008–2009 (cycle G) and in 2011–2012 (cycle H). While LASA has been collecting data since 1992, we only included these cycles because visual acuity was only measured at these time points.

### Participants

LASA’s first cohort was formed in 1992 from a random sample of people aged 55 to 85 years, drawn from population registers in 11 municipalities in the Netherlands. The acquired sample was stratified for age, gender and level of urbanisation. This sample was first used in the NESTOR study on Living Arrangements and Social Networks (LSN) [40]. In 2002–2003, a second cohort was formed from an identical sampling frame. This process has shaped representative samples of the older Dutch population and has further been described in detail in previous publications [41–43]. In total, data were collected on 2599 unique participants. Selection bias was kept to a minimum by including a very large population, by recruiting participants from three culturally distinct areas with different levels of urbanisation and by contacting members of a general population rather than clinical recruitment [44].

### Outcome measures

#### Visual acuity

Visual acuity was reported in terms of visual acuity rating (VAR), measured using a Colenbrander 1-m chart with a + 1.00 dioptre magnifying glass [45]. For analysis, all obtained VAR scores were converted to log units (logarithm of the Minimal Angle of Resolution, logMAR) [46]. Visual acuity of the better eye was used for analysis, regardless of lateralisation.

#### Mental health

Validated Dutch translations of widely-used questionnaires were deployed to assess different aspects of mental health. For depressive symptoms, the Center for Epidemiologic Studies – Depression scale (CES-D) [47] was used. The CES-D questionnaire contains 20 items, measuring depressive symptoms on a 4-point Likert scale (scored 0–3). For symptoms of anxiety, the Hospital Anxiety and Depression Scale – Anxiety Subscale (HADS-A) [48] was used. The HADS-A contains 7 items, measuring symptoms of anxiety on a 4-point Likert scale (scored 0–3). For mastery, the Pearlin Mastery Scale (PMS) [30] was used. The PMS questionnaire contains 7 items, measuring mastery on a 5-point Likert scale (scored 0–4). For self-esteem, the Rosenberg Self-Esteem Scale (RSES) [49] was used. The RSES questionnaire contains 10 items, of which the first 4 were included, measuring self-esteem on a 5-point Likert scale (scored 0–4). Items using adverse wording were coded reversely. Thus, higher scores corresponded with greater levels of depression, anxiety, mastery and self-esteem, respectively.

#### Other variables

Additionally, other variables were obtained, including age, education, nationality, living arrangement (independent or not), marital status (currently married or not), partner status (partner being present or not), personal network size, functional limitations (i.e. dressing and undressing, chair use, clipping one’s toenails, walking, transportation and stair use), special housing adjustments (none or one and more) and chronic somatic comorbid diseases (i.e. chronic non-specific lung disease, cardiac disease, peripheral artery disease, stroke, diabetes mellitus, arthritis and malignancies). These variables were chosen either to describe essential characteristics of the research population, or because previous literature showed them to be factors of importance in the studied associations. During statistical analysis, age was found to grossly violate the linearity assumption, rendering the variable unfit to be included as a continuous measure. Therefore, age was divided into three groups to facilitate separate analysis for three clinically relevant groups: 1) a working-age population (up to 65 years old in the Netherlands), 2) a middle old population (65 to 90 years old) and 3) the oldest old (90 years and older). For the oldest old we chose a cut-off of 90 years and older since this is the fastest growing segment of the Dutch population and this age group constitutes a growing and distinct group of patients with visual impairment [50]. To address the possible issue of information bias, data collection on outcomes occurred in a highly structured fashion and similarly for all participants.

### Statistical analyses

#### Data preparation

IRT-analyses – also called *latent trait analyses* – were performed on the questionnaires at all measurement time-points to estimate individual latent trait (*θ*-)scores per item. These statistical models incorporate the characteristics of questionnaire items and all obtained responses, rendering a more accurate representation of the respondent’s score on the latent construct, which was originally set out to be measured. Item-response models provide certain compelling advantages, describing the relationship between a latent trait, the characteristics of the items in the scale and the answers of respondents to the individual items [51]. This results in a more accurate estimation of one’s true *latent trait* (e.g. level of depression), increasing the validity of the used questionnaires and the accuracy of the obtained results. A Graded Response Model (GRM) was chosen as the preferred IRT-model for its flexibility regarding item goodness-of-fit [52]. In order for IRT-analyses to be accurate, questionnaires should meet the criteria for three crucial assumptions; unidimensionality, local independence of items and monotonicity [53]. Unidimensionality was tested using standard indices. Local independence of items and monotonicity were checked by analysing residual covariance and plotting results of Mokken analyses, respectively [54]. Confirmatory Factor Analysis (CFA) was conducted to assess goodness-of-fit and the estimated number of fundamental factors in the model. Based upon the retrieved parameters, the acquired *θ* (ranging from − 4.0 to + 4.0) was used in further analysis. Data preparation was conducted in RStudio, Version 0.99.896.

#### Primary analyses

Linear mixed modelling (LMM) with maximum likelihood estimation was used to estimate the associations between logMAR visual acuity and mastery, self-esteem, depression and anxiety. LMM is a preferred statistical method for analysing longitudinal data, using random intercepts and fixed variables. This particular model was chosen for its superior properties in dealing with missing values – which were inherent to the design of the study – and its integration of both interpersonal and intrapersonal variance [55]. Possible confounding [44] was analysed and adjusted for. Analyses were carried out using logMAR visual acuity as a continuous independent measure for visual impairment. IBM SPSS Statistics, Version 22.0 was used to conduct the analyses.

#### Mediation analyses

We hypothesise that mastery and self-esteem mediate the effect between visual acuity (the independent variable) and depression and anxiety (dependent variables) over time. First, an LMM with maximum likelihood estimation was performed to describe the total effect of the independent variable on the dependent variables. Second, LMM analysis was performed to calculate the ‘a-path’; the association between the independent variable and the potential mediators. Third, a final LMM with maximum likelihood estimation was performed to estimate the direct effect (c’) of the independent variable on the dependent variables, whilst controlling for the potential mediators (b), by including it in the model. Subsequently, these three pathways were compared. Again, potential confounding was analysed and adjusted for.

## Results

### Participant characteristics

**Fig. 1:**
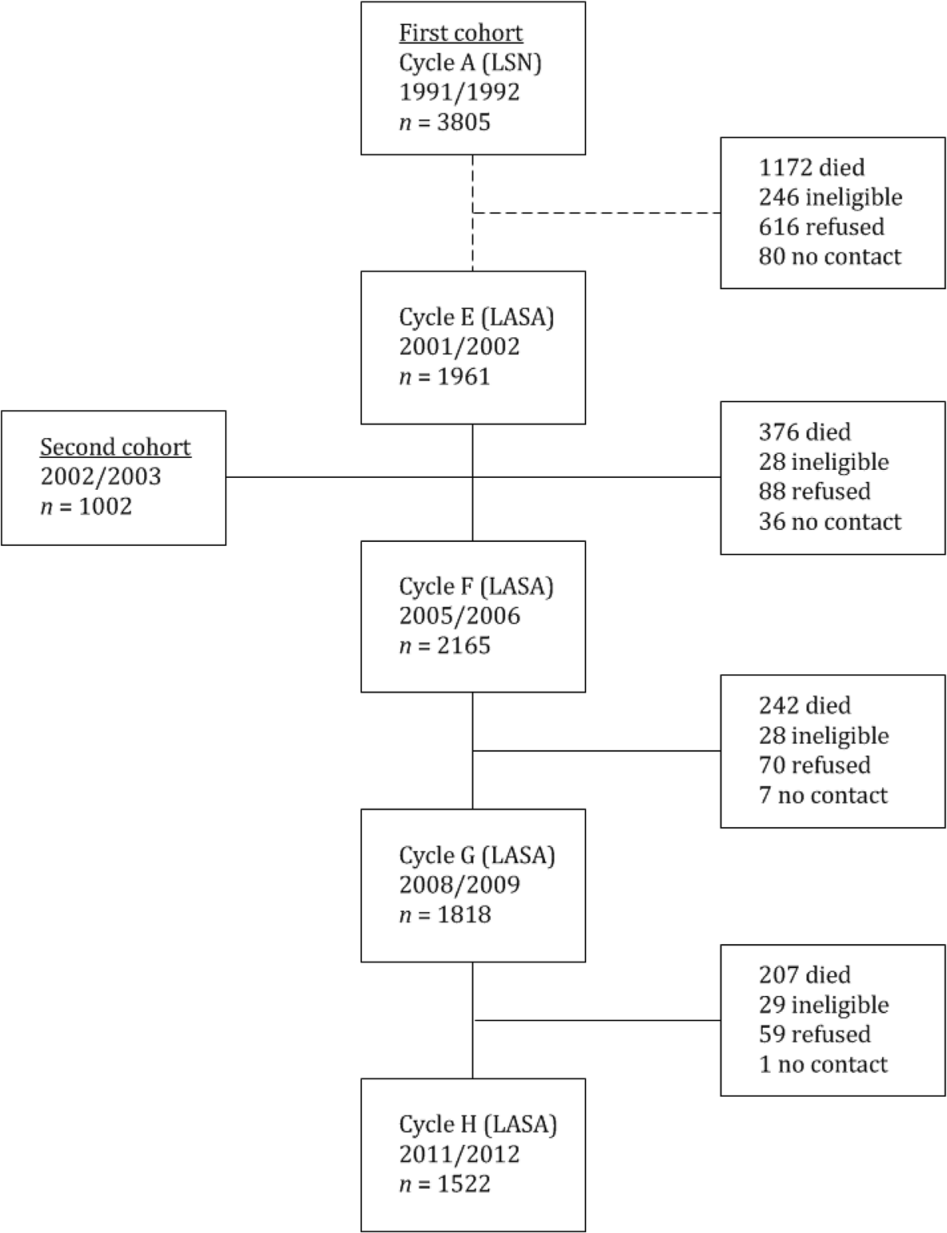
Inclusion, loss to follow up and attrition per measurement

Data pertaining to participant inclusion, follow-up rates and attrition was retrieved from previous LASA publications (see Fig. 1) [39]. Our study only included data that were collected between 2001 and 2012 (cycles E to H) because visual acuity was measured at these time points. Several participants dropped out during the course of the study because they died, were ineligible to participate, no longer wanted to participate or could not be contacted. Participants were deemed ineligible when they no longer met the initial inclusion criteria [56]. Due to this loss to follow-up and the inclusion of participants at various moments in time, the mean follow-up time was 5.9 years with a standard error of 0.07 years This was calculated using the difference between age at first measurement and age at last measurement for each participant. A baseline summary containing data extracted from participants’ first available measurement (*n* = 2599 in total, of which *n* = 1961 for cycle E and *n* = 638 for cycle F), is available in Table 1. Mean age was 72 years, 56% was female, almost all had the Dutch nationality, 72% had one or more chronic somatic diseases and 1.2% qualified as having low vision.

**Table 1:** Patient characteristics at baseline (total *n* = 2599 of which *n* = 1961 for cycle E and *n* = 638 for cycle F)

Independent variables	
Visual acuity (logMAR), mean (SE)	0.08 (0.003)
Visual Group†	
No visual impairment, *N* (%)	2020 (78%)
Low vision, *N* (%)	32 (1,2%)
Missing, *N* (%)	547 (21%)
**Dependent variables ҂**	
Depression (*θ*), mean (SE)	0.0044 (0.0186)
Anxiety (*θ*), mean (SE)	0.086 (0.0170)
Mastery (*θ*), mean (SE)	0.046 (0.0192)
Self-esteem (*θ*), mean (SE)	− 0.00122 (0.0186)
**Other variables**	
Age, mean (SE)	72 (0.181)
Age Group	
Up to 65 years, *N* (%)	682 (26.2%)
65 to 90 years, *N* (%)	1814 (70%)
90 years and up, *N* (%)	103 (4.0%)
Female gender, *N* (%)	1457 (56%)
Living independently, *N* (%)	2251 (87%)
Currently married, *N* (%)	1573 (61%)
Dutch nationality, *N* (%)	2585 (99%)
Network size, mean (SE; range)	16.1 (0.200; 0–67)
Number of chronic somatic disorders	
None, *N* (%)	650 (25%)
One, *N* (%)	892 (34%)
Two or more, *N* (%)	783 (30%)
Missing, *N* (%)	274 (11%)
No functional limitations, *N* (%)	1075 (41%)
Having a partner, *N* (%)	1683 (65%)
No special housing adjustments, *N* (%)	1709 (66%)

† Low vision was defined as logMAR visual acuity of 0.50 and higher

҂ Factor scores (range: −4.0 to 4.0), representing latent trait scores, acquired by Item Response Theory (IRT-)analysis on the used questionnaires – for depression; *CES-D* (Center for Epidemiologic Studies – Depression Scale) [29], *HADS-A* (Hospital Anxiety and Depression Scale – Anxiety Subscale) [30], *PMS-5* (5-item Pearlin Mastery Scale) [31] and *RSES* (Rosenberg Self-Esteem Scale) [32]

### Data preparation

IRT-analyses were performed on the questionnaires for depression, anxiety, mastery and self-esteem at all four measurement time-points to estimate individual latent trait (θ-)scores per item. The preferred indices to report goodness-of-fit of these models include the Comparative Fit Index (CFI), the Tucker Lewis Index (TLI) (also called the Non-Normed Fit Index (NNFI)) and the Root Mean Square Error of Approximation (RMSEA). According to various experts in this field, a fair to good fit is indicated by CFI- and TLI-values of 0.9 and higher and a RMSEA-value of 0.07 or lower [57, 58]. In Table 2, the mean scores of these values, as calculated over the four measurements in time, are reported. All questionnaires showed a good fit based on the CFI, TLI/NNFI and RMSEA. A lack-of-fit was only found for the 7-item Pearlin Mastery Scale (PMS). Two items performed poorly, violating both the monotonicity and unidimensionality assumptions. During principal components analysis, second factor loadings were remarkably high for these items. The items were therefore omitted, one of which was disregarded in previous research as well because of redundancy [59]. This resulted in the 5-item mastery scale (‘PMS-5’), which was used during further analysis, with evidently better performance on all indices, most importantly a decrease of the Root Mean Square Error of Approximation (RMSEA) from 0.106 to 0.078.

**Table 2:** Goodness-of-fit indices for the used questionnaires

	CFI	TLI/NNFI	RMSEA
**Depression (CES-D)**	0.97	0.97	0.067
**Anxiety (HADS-A)**	0.99	0.99	0.053
**Mastery (PMS)**	*0.96*	*0.95*	*0.106*
Mastery (PMS-5)	0.99	0.98	0.078
**Self-esteem (RSES)**	1.00	1.00	0.0148

Center for Epidemiologic Studies – Depression Scale (*CES-D*), Hospital Anxiety and Depression Scale – Anxiety Subscale (*HADS-A*), Pearlin Mastery Scale (*PMS*) and Rosenberg Self-Esteem Scale (*RSES*)

### Primary analyses

For LMM analyses based on continuous logMAR visual acuity data, regression coefficients, standard errors and *p*-values are summarised in Table 3. In the uncorrected models, visual acuity was associated with significantly lower depression scores (*β* = 0.341, *p* < 0.001) and a greater sense of mastery (*β* = − 0.589, *p* < 0.001) and self-esteem (*β* = − 0.215, *p* < 0.001). No significant association between visual acuity and anxiety was found.

**Table 3:** Linear mixed models (LMMs) on logMAR visual acuity

	Dependent variables						Potential mediators					
	Depression (CES-D)			Anxiety (HADS-A)			Mastery (PMS-5)			Self-esteem (RSES)		
	*β*	SE	*p*	*β*	SE	*p*	*β*	SE	*p*	*β*	SE	*p*
**Visual Impairment**												
Crude analysis	**0.341**	0.059	0.000	0.082	0.057	0.147	**−0.589**	0.066	0.000	**−0.215**	0.062	0.000
Adjusted analysis ^+^	**0.235**	0.060	0.000	0.032	0.058	0.579	**−0.477**	0.067	0.000	**−0.166**	0.063	0.008

^+^ Adjusted for gender, age and number of comorbid chronic disorders

*CES-D* (Center for Epidemiologic Studies – Depression Scale) [29], HADS-A (Hospital Anxiety and Depression Scale – Anxiety Subscale) [30],

*PMS-5* (5-item Pearlin Mastery Scale) [31] and *RSES* (Rosenberg Self-Esteem Scale) [32]

Models were checked for confounding on demographic variables, and gender, age and number of comorbid chronic diseases had a significant influence. Therefore, all models were adjusted for gender, age and comorbid diseases (See Table 3). In the corrected models, logMAR visual acuity was associated with significantly higher depression scores (*β* = 0.235, *p* < 0.001) and a lower sense of mastery (*β* = − 0.477, *p* < 0.001) and self-esteem (*β* = − 0.166, *p* = 0.008). Still, no significant association between visual acuity and anxiety was found.

### Mediation analyses

Since no significant association between visual acuity and anxiety was found, the mediated effect of mastery and self-esteem was only investigated in the association between visual acuity and depression.

With self-esteem included as a potential mediator (see Table 4), the total effect of visual acuity on depression diminished from β = 0.341 to a direct effect of β = 0.271 in the uncorrected model and from β = 0.235 to β = 0.164 in the corrected model. This amounts to a mediated percentage of 22.0% for the uncorrected model and 25.4% for the corrected model. The direct effect of visual acuity on depression remained statistically significant in the final model.

**Table 4:** Linear mixed models (LMMs) on the mediation of self-esteem and mastery in the association between visual acuity and depression

			Crude model			Adjusted model ^+^		
			*β*	SE	*p*	*β*	SE	*p*
**Depression**								
Total effect	***c***	Visual acuity	**0.341**	0.059	0.000	**0.235**	0.060	0.000
*a*-path	***a***	Visual acuity	**−0.215**	0.062	0.001	**−0.166**	0.063	0.008
Direct effect	***c’***	Visual acuity	**0.271**	0.060	0.000	**0.164**	0.061	0.007
	***b***	Self-esteem	**−0.355**	0.0128	0.000	**−0.336**	0.0128	0.000
**Depression**								
Total effect	***c***	Visual acuity	**0.341**	0.059	0.000	**0.235**	0.060	0.000
*a*-path	***a***	Visual acuity	**−0.600**	0.067	0.000	**−0.458**	0.063	0.000
Direct effect	***c’***	Visual acuity	**0.124**	0.059	0.032	0.054	0.060	0.366
	***b***	Mastery	**−0.388**	0.013	0.000	**−0.366**	0.013	0.000

*β*-estimates printed in boldface when significant at the *p* < 0.05 level

^+^ Adjusted for gender, age and number of comorbid chronic disorders

With mastery included as a potential mediator (see Table 4), the total effect of visual acuity on depression diminished from β = 0.341 to a direct effect of β = 0.124 in the uncorrected model and from β = 0.235 to β = 0.047 in the corrected model. This amounts to a mediated percentage of 65% for the uncorrected model and 79% for the corrected model. The direct effect of visual acuity on depression lost its statistical significance in the final model.

## Discussion

Visual impairment was found to be associated with depression over time. Previous longitudinal studies reported conflicting results: some found an association between visual impairment and the development of depression [13, 60–65], while others did not [66, 67]. We provide additional evidence based on a large sample and longitudinal follow-up supporting the hypothesis that visual impairment predicts depression. This accentuates the importance of adequate detection of mental health changes in those who develop visual impairment over time. Therefore, screening and monitoring procedures for depression should be a routine part of low vision care.

Still, some individuals with severe vision loss may experience no depressive symptoms, while others with minor vision loss can be severely depressed and symptoms can fluctuate over time [68]. Identification of intrapersonal factors responsible for these variations are therefore essential to understand the impact of visual impairment on mental health and its consequences on offering tailored support. In our study we found that the association of visual impairment and depression is mediated by loss of self-esteem and loss of mastery, which is supported by recent literature [69, 70]. People with visual impairment often feel a loss of control [33–35] and reduced self-esteem [38] in performing activities in everyday life. These intrapersonal factors largely explain the development of depression in this population. By addressing these factors in healthcare, we may be able to approach the imminent problem of depression in patients with visual impairment. In addition, it may help identify those who are at risk of developing depression and to be able to intervene at an earlier stage. Prior studies suggest that offering group-based rational emotive behavioural therapy within low vision service settings may improve self-esteem in people with visual impairment [71], which may in turn reduce depression. Moreover, a self-management program [72, 73] and a social competence training [74] offered within low vision services may increase self-efficacy in this population. Self-efficacy, i.e. believing in one’s own ability to produce desirable results in a specific area, is related to mastery. People with high self-efficacy about a certain task will most likely have a high sense of mastery. Still, a recent meta-analysis showed that the certainty of evidence on interventions to improve self-efficacy and self-esteem in adults with visual impairment is low with a high risk of bias, high risk of imprecision and inconsistency in results [75]. Therefore, more high quality studies are needed to improve evidence-based care to address mastery and self-esteem in people with visual impairment, and in turn reduce depression.

In apparent contrast to results from previous cross-sectional [10, 76] and longitudinal studies [60, 77], an association between vision loss and anxiety was not found. A recent longitudinal cohort study in older adults (*n* = 7584) by Frank et al. also found no association between self-reported visual impairment and anxiety [64]. Differences may be explained by the way visual impairment is measured. Vision loss encompasses more than just visual acuity (e.g. visual field defects) and self-reported (subjective) vision loss as a measurement for visual impairment may potentially invite response bias (e.g. recall and social desirability bias) and confounding by personality type [64, 78]. Additional investigations are needed to confirm findings and establish whether a causal association exists.

### Strengths and limitations

The use of longitudinal data from a large general population has augmented this study’s value. Most notably, this research has contributed to visual impairment research by unravelling the mediating roles of self-esteem and mastery in the longitudinal association between visual acuity and depression, which has not previously been attempted. In addition, IRT analyses were performed to increase the accuracy of the results based on validated questionnaires. Because of the large and representative sample size and the broad study design, our findings may be generalizable to other community-dwelling populations in high-income countries. However, for countries fundamentally dissimilar to the Netherlands, the strength of the found associations might be different.

Also, visual impairment has a relatively low prevalence in the investigated general population. Moreover, visual acuity is but a partial measure for visual functioning. For example, the integrity of the visual field, high-contrast dependency but also experienced visual disability have not been taken into account. Future studies may attempt to incorporate these aspects to more fully assess visual functioning in relation to mental health. In addition, we were not able to analyse and control for possible confounding on the time of onset of the visual impairment, activities of daily living and hearing impairment, which may have played an additional role in the association between visual acuity and mental health.

## Conclusion

In our longitudinal cohort study (*n* = 2599), better visual acuity was associated with greater sense of mastery and self-esteem, and less depression. The relationship between visual acuity and depression was mediated by self-esteem (25%) and mastery (79%). These intrapersonal factors can be addressed in mental health programs to ultimately reduce depression.

## Data Availability

The datasets used and/or analysed during the current study are available from the corresponding author on reasonable request.

## Abbreviations

LASA: Longitudinal Aging Study Amsterdam
IRT: Item Response Theory
PST: Problem-solving treatment
LSN: Living Arrangements and Social Networks
METc: Medical Ethics committee
VAR: Visual acuity rating
logMAR: Logarithm of the Minimal Angle of Resolution
CES-D: Center for Epidemiologic Studies – Depression scale
HADS-A: Hospital Anxiety and Depression Scale – Anxiety subscale
PMS: Pearlin Mastery Scale
RSES: Rosenberg Self-Esteem Scale
LMM: Linear mixed modelling
GRM: Graded Response Model
CFA: Confirmatory Factor Analysis
RMSEA: Root Mean Square Error of Approximation
